# Rift Valley Fever: A survey of knowledge, attitudes, and practice of slaughterhouse workers and community members in Kabale District, Uganda

**DOI:** 10.1371/journal.pntd.0006175

**Published:** 2018-03-05

**Authors:** Annabelle de St. Maurice, Luke Nyakarahuka, Lawrence Purpura, Elizabeth Ervin, Alex Tumusiime, Stephen Balinandi, Jackson Kyondo, Sophia Mulei, Patrick Tusiime, Craig Manning, Pierre E. Rollin, Barbara Knust, Trevor Shoemaker

**Affiliations:** 1 Centers for Disease Control and Prevention, Division of High Consequence Pathogens and Pathology, Viral Special Pathogens Branch, Atlanta, GA, United States of America; 2 Department of Arbovirology, Emerging and Re-emerging Disease, Uganda Virus Research Institute, Entebbe, Uganda; 3 Makerere University, College of Veterinary Medicine, Animal Resources and Biosecurity, Department of Biosecurity, Ecosystems and Veterinary Public Health, Kampala, Uganda; 4 Kabale District Health Office, Kabale, Uganda; University of Texas Medical Branch, UNITED STATES

## Abstract

**Background:**

Rift Valley Fever virus (RVF) is a zoonotic virus in the *Phenuiviridae* family. RVF outbreaks can cause significant morbidity and mortality in humans and animals. Following the diagnosis of two RVF cases in March 2016 in southern Kabale district, Uganda, we conducted a knowledge, attitudes and practice (KAP) survey to identify knowledge gaps and at-risk behaviors related to RVF.

**Methodology/Principal findings:**

A multidisciplinary team interviewed 657 community members, including abattoir workers, in and around Kabale District, Uganda. Most participants (90%) had knowledge of RVF and most (77%) cited radio as their primary information source. Greater proportions of farmers (68%), herdsmen (79%) and butchers (88%) thought they were at risk of contracting RVF compared to persons in other occupations (60%, p<0.01). Participants most frequently identified bleeding as a symptom of RVF. Less than half of all participants reported fever, vomiting, and diarrhea as common RVF symptoms in either humans or animals. The level of knowledge about human RVF symptoms did not vary by occupation; however more farmers and butchers (36% and 51%, respectively) had knowledge of RVF symptoms in animals compared to those in other occupations (30%, p<0.01). The use of personal protective equipment (PPE) when handling animals varied by occupation, with 77% of butchers using some PPE and 12% of farmers using PPE. Although most butchers said that they used PPE, most used gumboots (73%) and aprons (60%) and less than 20% of butchers used gloves or eye protection when slaughtering.

**Conclusions:**

Overall, knowledge, attitudes and practice regarding RVF in Kabale District Uganda could be improved through educational efforts targeting specific populations.

## Author summary

Rift Valley Fever (RVF) virus is transmitted to humans from contact with infected livestock and through mosquito bites. Several human cases of RVF were diagnosed in Kabale District, Uganda in March 2016, over 40 years after the last RVF case was identified in Uganda. We administered a knowledge, attitudes, and practice survey to people living in Kabale District, near where the cases occurred. Survey results demonstrated that knowledge, attitudes and practice surrounding RVF could be improved within the community.

## Introduction

Rift Valley Fever (RVF) virus is a single-stranded negative sense RNA virus of the genus *Phlebovirus* in the *Phenuiviridae* family [1,2]. RVF virus outbreaks can cause significant morbidity and mortality in animals and humans [3]. In sheep, goats, and cattle, infection with RVF can lead to increased abortions and stillbirths. RVF symptoms in humans range from asymptomatic or a mild flu-like illness to a more severe illness including hepatitis, retinitis, or encephalitis; approximately 1% of human cases develop hemorrhagic disease [4]. Case fatality is estimated at 1–2% [5], however during an outbreak in Saudi Arabia, case fatality was estimated to be as high as 17% [6]. RVF virus is maintained in infected mosquitoes, and transmitted to ruminants including cows, sheep, and goats via mosquito bites [3]. Mosquito vectors that have been implicated in RVF transmission include species from six genera: Aedes, Culex, Anopheles, Eretmapodites, Mansonia, and Coquilletidia [3,7]. These vectors have been identified in regions spanning the African continent [7]. Experimental models have demonstrated that North American mosquito species are competent vectors [8,9]. Humans primarily become infected with RVF from contact with blood or body fluids of infected animals through slaughtering, caring for sick animals, or assisting with animal birth. As a result, herdsmen and butchers are at increased risk of RVF infection due to exposure to blood and body fluids of infected animals [10–12]. Mosquito bites are another source of RVF transmission to humans. Consumption of raw meat and dairy is not a confirmed mode of RVFV transmission but is a risk factor for other zoonotic diseases such as brucellosis and listeria.

In agricultural communities, RVF outbreaks can cause significant economic losses [3]. The 2007 RVF outbreak in Kenya impacted agricultural production and employment, resulting in an estimated loss of $32 million USD, due to both direct effects from livestock death and indirect effects including reduced income from the closure of livestock markets and reduced sales of animal derived products [13]. Outbreaks typically occur after periods of heavy rainfall and flooding lead to increased mosquito populations; therefore it is possible to use geospatial analysis to predict areas with increased risk of RVF outbreaks [14–16]. Early identification of cases in livestock and humans is an important tool in outbreak detection and may aid in the rapid deployment of disease control measures, such as animal vaccines or mosquito control. There are no approved RVF vaccines for humans. Therefore, the main methods of disease prevention in people are early detection, awareness, and behavior modification such as the use of personal protective equipment and increased hand hygiene. Therefore it is important to ensure that at-risk populations in endemic regions are aware of RVF.

In March 2016, two human RVF cases were confirmed in Kabale District, Uganda [17]. These were the first RVF cases identified in Uganda since 1968 [18]. Following the identification of these two cases, an investigation was coordinated by the Uganda Ministry of Health, Uganda Ministry of Agriculture Animal Industry and Fisheries (MAAIF), the Uganda Virus Research Institute (UVRI), and United States Centers for Disease Control and Prevention (CDC) to respond locally to the outbreak and to assess the knowledge, attitudes, and practices (KAP) of community members living in the area. The main objective of a KAP study is to identify knowledge gaps in order to create programs and materials to address those needs. Although RVF KAP studies have been performed previously in Eastern Africa [19–23], we could not find published RVF KAP studies performed in Uganda.

## Methods

### Setting

Kabale District is located in the southwest corner of Uganda. According to the 2014 Uganda Census, Kabale had an estimated population of 534,160 people, with the majority living in a rural setting (457,592/534,160; 86%) [24]. The altitude of Kabale ranges from 1,219 m to 2,347 m above sea level. Agriculture is an important source of revenue in this region. Corn, beans, potatoes, bananas, millet, coffee, apples, and tea are all grown in the region. Livestock also provide a source of income for families through the sale of meat and dairy. Most families own goats, however sheep, cattle, and pigs are also common. In addition to agricultural lands, Kabale District also has areas with high altitude forest and savannahs.

### Participant recruitment strategy

**Fig 1:**
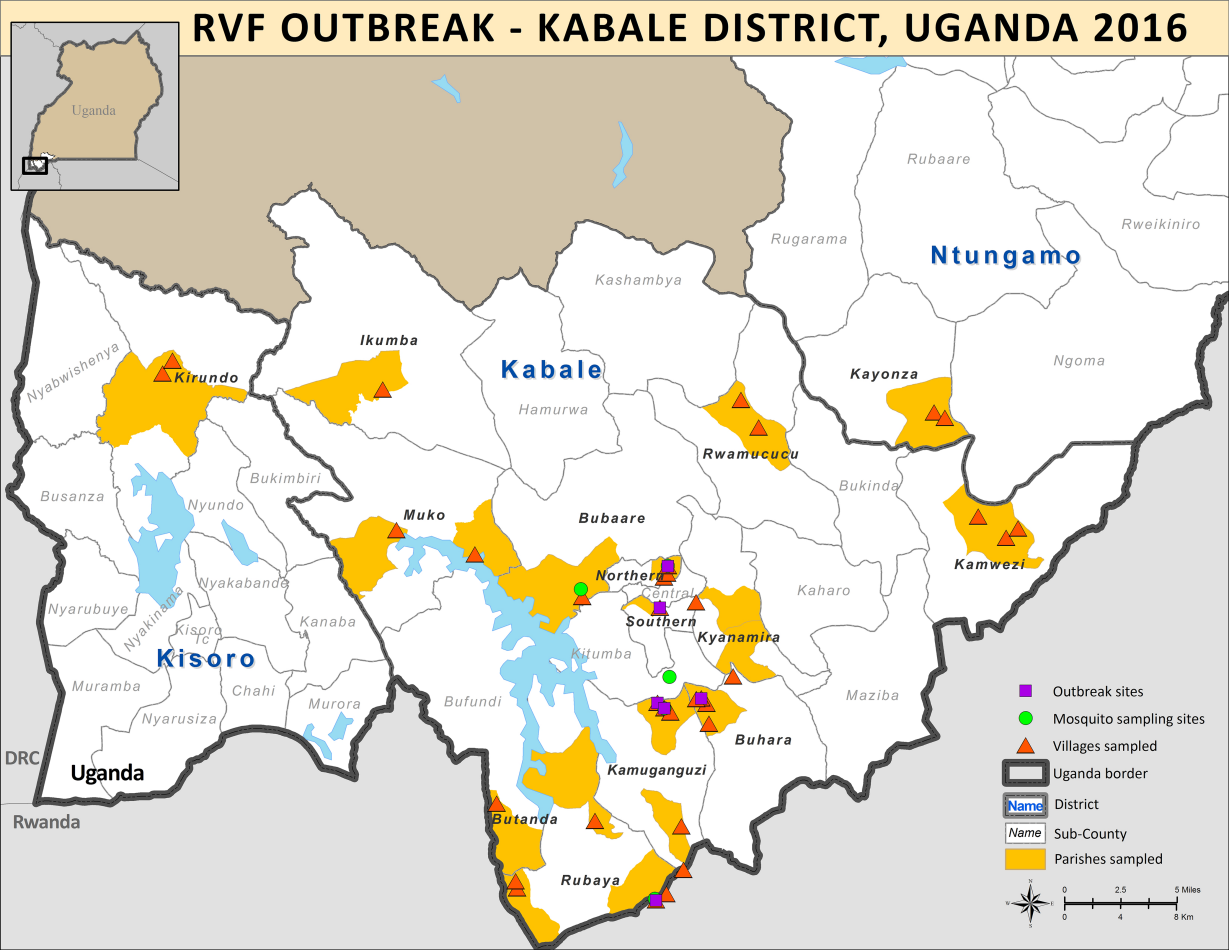
Map of study region. Purple squares represent the site of a probable or confirmed case in the outbreak; and the yellow indicates parishes sampled in Kabale District as part of the KAP assessment. The red triangles represent the villages that were sampled. The Green circles represent sites where mosquitoes were sampled for a separate RVF study. *This figure was created specifically for this manuscript in ArcGIS using open source data from ESRI and DIVA-GIS for the background layers*, *and GPS points collected in the field*.

A multidisciplinary team, consisting of individuals from the Uganda Ministry of Health, Uganda MAAIF, UVRI, and CDC, visited 34 sites from April 1–12 2016 in Kabale District, Uganda to recruit participants to complete a RVF KAP cross-sectional survey (Fig 1). Recruitment sites included the main abattoir in the town/city of Kabale, villages where a human RVF case had been identified, and areas where no human cases had been identified previously. The team arrived at a site and local health workers recruited participants from the village. Convenience sampling was used; all participants who arrived at the site were eligible for the study. Children older than seven years were allowed to participate if a parent provided written consent.

### Questionnaire

The survey was written in English but was read aloud in a private setting to participants by trained interviewers in the local language. The survey questionnaire asked participants about sociodemographic variables as well as epidemiological risk factors and exposures, including contact with animals and exposure to raw milk, raw meat, and mosquitoes (S1 Fig).

Occupation was self-reported and individuals described their primary occupation as a farmer, herdsman, butcher, or another occupation. Interviewers also asked about the use of personal protective equipment (e.g. masks, gloves, gumboots) when in contact with animals. Participants were asked questions about knowledge of RVF symptoms, transmission, and prevention. Participants answered questions about their attitudes towards the existence of RVF, welcoming RVF survivors into their community, and health seeking practices. Most questions were closed-ended. However, participants were asked open-ended questions about why they did not use a mosquito net, about fears and risks of contracting disease, methods to protect themselves from disease, and ways to prevent RVF transmission.

### Analysis

Data were analyzed using STATA 13.0 (StataCorp. 2013. *Stata Statistical Software*: *Release 13*. College Station, TX: StataCorp LP.). Differences in frequency distribution between groups were compared using Pearson’s chi-square for categorical variables. Results were considered statistically significant if the p-value was <0.05.

### Ethical considerations

Approval for the study was obtained from UVRI Research Ethics Committee and the CDC Human Subjects Advisor group. The CDC National Center for Emerging and Zoonotic Infectious Diseases (NCEZID) Human Subjects Advisor group determined that this was part of an outbreak investigation and was classified as non-research. The NCEZID Human Subjects Advisory group was aware of the study protocol and consent process. Participants completed a consent form for the questionnaire that was translated from English into local languages by team members. Parents of minors consented to participation prior to interviewing. All participation was voluntary and participants did not receive any compensation.

## Results

### Participant demographics

A total of 657 participants were interviewed, most (40%) were males aged 20–49 years (Table 1). The mean age of participants was 40 years, with a range of 7 years to 90 years. Most participants were from sites where no RVF case had been identified (293/647; 45%). In addition, 238 (37%) of participants were recruited from sites where a previous RVF case had been identified, and 117 (18%) of participants were butchers who worked at the main abattoir in Kabale. Most participants owned domestic animals and had contact with goats (353/504; 70%), cattle (299/505; 59%), pigs (129/505; 26%), sheep (98/506; 19%), and poultry (91/505; 18%). Participants who owned livestock, had on average three cattle, four goats and four sheep. Few participants had contact with ducks (3/506; 0.6%) or rabbits (9/506; 2%).

**Table 1:** Participant demographics.

Variable	Female n(% of Female)	Male n(% of Male)	P-value*	Total Number N (%)**
**Number individuals**	218 (34%)	428 (66%)		657
**Age group**				
7–19 years	14 (6%)	50 (12%)	**0.03**	66 (10%)
20–49 years	135 (62%)	258 (60%)	0.7	397 (60%)
≥50 years	69 (32%)	120 (28%)	0.3	194 (30%)
**Education N(%)**				
None	24 (11%)	112 (26%)	**<0.01**	154 (23%)
Primary	111 (51%)	248 (58%)	0.09	141 (22%)
Secondary and Post-Secondary	83 (38%)	68 (16%)	**<0.01**	362 (55%)
**Marital Status N(%)**				
Unmarried	61 (30%)	102 (26%)	0.3	167 (27%)
Married	144 (70%)	293 (74%)		442 (73%)
**Occupation N(%)**				
Herdsman	2 (1%)	31 (7%)	**<0.01**	33 (5%)
Farmer	92 (42%)	130 (30%)	**<0.01**	228 (35%)
Butcher	1 (0.5%)	114 (27%)	**<0.01**	115 (18%)
Other	123 (56%)	153 (36%)	**<0.01**	246 (37%)
**Own Domestic Animals**				
No	108 (50%)	152 (36%)	**<0.01**	264 (40%)
Yes	109 (50%)	276 (64%)		392 (60%)
**Contact with Animals**				
No	76 (35%)	69 (16%)	**<0.01**	147 (22%)
Yes	142 (65%)	359 (84%)		510 (78%)

*Comparing males to females using chi-square

**Total column includes respondents who did not have a gender specified.

Participants most frequently had contact with live animals during grazing (Table 2).

**Table 2:** Types of contact with animals and animal products.

Type of Animal Contact	Female n (% of Female)	Male n (% of Male)	P-value*	Total n(%)**
**Live Animal Contact (n = 506)**				
Milking	11 (7.8%)	60 (17%)	**<0.01**	72 (14%)
Grazing	128 (91%)	218 (61%)	**<0.01**	354 (70%)
Grooming	45 (32%)	82 (23%)	**0.04**	131 (26%)
Caring for sick	25 (18%)	88 (25%)	0.09	113 (22%)
Assisting with birth	24 (11%)	182 (44%)	**<0.01**	211 (33%)
Sleeping near animals	18 (13%)	44 (12%)	0.9	64 (13%)
**Dead Animal Contact**				
Slaughtering/Butchering (respondents = 519)	17 (12%)	219 (60%)	**<0.01**	238 (46%)
Handling raw meat (respondents = 561)	143 (83%)	284 (75%)	**0.03**	444 (77%)
**Eating practices**				
Drinking raw milk	5 (2.3%)	31 (7.3%)	**<0.01**	36 (6%)
Eating raw meat	6 (2.8%)	24 (5.6%)	**<0.01**	30 (5%)

*Comparing males to females using chi-square

**Total column includes respondents who did not have a gender specified.

A third (33%) of participants also reported assisting with animal birth. Contact with dead animals was mainly through handling raw meat (77%). However, nearly half of participants said that they were involved in slaughtering or butchering (Table 2). Significantly more men than women were engaged in milking, birth assistance and slaughtering/butchering (Table 2). However, significantly more women assisted with animal grazing (Table 2). Few participants drank raw milk or ate raw meat.

### Participant knowledge of RVF

Most (90%) of participants said that they had heard of RVF previously; a greater proportion of butchers (95%), herdsmen (94%), and farmers (92%), had heard of RVF compared to persons in other occupations (85%) (X^2^ = 10.4; p = 0.016). Most respondents had heard of RVF from the radio (77%).

Interviewers asked participants if they had knowledge of RVF symptoms in humans and animals, participants responded either “yes, no, or do not know” (S1 Fig). Most participants, regardless of their occupation, said that they could identify RVF symptoms in humans (Table 3 and Table 4). Clinical symptoms of RVF are listed in Table 3. The most common human symptom identified by participants was bleeding. Less than half of participants cited fever, vomiting, or diarrhea as RVF symptoms in humans. Of all participants, 241 (37%) had knowledge of RVF symptoms in animals (Table 3). Knowledge of RVF symptoms in animals was significantly higher among herdsmen and butchers. Bleeding in animals was the most common RVF symptom reported by participants, particularly among butchers, farmers, and individuals in other occupations. Herdsmen were significantly more likely to identify fever as an RVF symptom in animals. In contrast, butchers were more likely to identify nasal discharge as an RVF symptom in animals.

**Table 3:** Participant knowledge of RVF symptoms by occupation.

	Total Population* n (% of Total)	Farmers n (% of Farmer)	Herdsmen n (% of Herdsmen)	Butchers n (% of Butchers)	Other n (% of Other)	P-value**
**Any Knowledge of RVF Symptoms in Humans**	451 (69%)	151 (67%)	26 (79%)	84 (73%)	186 (68%)	0.4
**Signs and symptoms in Humans**						
Fever (respondents = 451)	191 (42%)	55 (36%)	11 (42%)	32 (38%)	40 (49%)	0.2
Bleeding (respondents = 454)	394 (87%)	131 (86%)	22(85%)	75 (89%)	164 (88%)	0.8
Vomiting (respondents = 452)	183 (40%)	70 (46%)	11 (42%)	34 (40%)	65 (35%)	0.2
Diarrhea (respondents = 452)	106 (23%)	41 (27%)	5 (19%)	18 (21%)	40 (22%)	0.6
**Any Knowledge of RVF Symptoms in Animals**	241 (37%)	81 (36%)	15 (45%)	59 (51%)	83 (30%)	**<0.01**
**Signs and symptoms in Animals**						
Bleeding (respondents = 241)	131 (54%)	45 (55%)	4 (27%)	40 (68%)	40 (49%)	**0.02**
Fever (respondents = 241)	71 (29%)	21 (26%)	9 (60%)	13 (22%)	25 (30%)	**0.03**
Nasal discharge (respondents = 241)	70 (29%)	19 (23%)	4 (27%)	26 (44%)	18 (22%)	**0.01**
Diarrhea (respondents = 241)	32 (13%)	14 (17%)	0 (0%)	8 (14%)	9 (11%)	0.3
Abortion (respondents = 241)	97 (40%)	31 (38%)	3 (20%)	27 (46%)	33 (40%)	0.3
Decreased milk production (respondents = 241)	4 (2%)	1 (1%)	0	0	1 (1%)	0.8
Prostration (respondents = 241)	13 (5%)	5 (6%)	0	3 (5%)	4 (5%)	0.8
Appetite loss (respondents = 241)	25 (10%)	9 (11%)	2 (13%)	6 (10%)	6 (7%)	0.8

*Total column includes respondents who did not have an occupation specified.

**Comparing by occupation using chi-square

**Table 4:** Participant knowledge of RVF symptoms by gender.

	Females n (% of Female)	Males n (% of Male)	P-value*	Total Population** n (%)
**Knowledge of RVF Symptoms in Humans** (respondents = 641)	148 (68%)	298 (70%)	0.6	451 (69%)
**Signs and symptoms in Humans**				
Fever (respondents = 451)	55 (37%)	135 (45%)	0.09	191 (42%)
Bleeding (respondents = 454)	138 (92%)	252 (84%)	**0.02**	394 (87%)
Vomiting (respondents = 452)	69 (46%)	112 (38%)	0.08	183 (40%)
Diarrhea (respondents = 452)	37 (25%)	68 (23%)	0.6	106 (23%)
**Knowledge of RVF Symptoms in Animals** (respondents = 652)	64 (29%)	174 (41%)	**0.02**	241 (37%)
**Signs and symptoms in Animals**				
Bleeding (respondents = 241)	33 (50%)	96 (56%)	0.4	131 (54%)
Fever (respondents = 241)	25 (38%)	45 (26%)	0.08	71 (29%)
Nasal discharge (respondents = 241)	13 (20%)	57 (33%)	**0.04**	70 (29%)
Diarrhea (respondents = 241)	5 (7.6%)	26 (15%)	0.1	32 (13%)
Abortion (respondents = 241)	28 (42%)	68 (40%)	0.7	97 (40%)
Decreased milk production (respondents = 241)	1 (1.5%)	3 (1.7%)	0.9	4 (2%)
Prostration (respondents = 241)	2 (3.0%)	11 (6.4%)	0.3	13 (5%)
Appetite loss (respondents = 241)	5 (7.6%)	20 (12%)	0.4	25 (10%)

*Compared proportions by gender using chi-square

**Total column includes respondents who did not have a gender specified.

In general, knowledge of RVF symptoms did not vary significantly by gender (Table 4). However, more women than men identified bleeding as a sign of RVF in humans. Significantly more men stated that they were familiar with RVF symptoms in animals and significantly more men identified nasal discharge as a symptom in animals.

Of all participants, 53% (345/655) said that they had any knowledge about how RVF is transmitted to humans or animals. Participants most frequently identified animal contact as a mode of transmission (269/348; 77%). We asked participants if RVF could be transmitted by human to human bodily contact (e.g. handshaking) and through contact with human bodily fluids (e.g. blood). Of all participants who responded, 83 of 348 (24%) said RVF could be transmitted through mosquitoes and through contact with body fluids of an infected person (84/348). Of farmers, 76 of 115 (66%) identified animals as a source of transmission, compared to 80–93% of butchers, herdsmen, and persons in other occupations (p = 0.003). Some participants thought that RVF could be transmitted by human-to-human bodily contact (107/348; 31%) and through air (25/348; 7%). The belief that RVF could be transmitted by bodily contact was most common among farmers (40%), compared to butchers (20%), herdsmen (20%), and others (29%) (p = 0.021). Most participants identified goats (267/288; 93%), cattle (260/290; 90%), and sheep (203/289; 70%) as sources of RVF transmission. Although our survey did not specifically ask whether or not eating raw meat or drinking raw milk were risk factors, 38 participants mentioned these as other possible sources of RVF transmission.

Our questionnaire also asked about ways to prevent RVF transmission; most participants cited avoiding animals (257/612; 42%) and sick people (122/612; 20%) as modes of prevention. A small proportion of respondents (92/612, 15%) cited sleeping under a mosquito net as a method of prevention. Although our questionnaire did not specifically ask about boiling milk and cooking meat, participants frequently mentioned them as possible methods to prevent RVF transmission.

### Participant practices surrounding RVF

Mosquito nets were used by most participants (82%; 539/655). Of those 116 individuals who did not use a mosquito net, the most frequently cited reason was that nets were uncomfortable, which was cited by 17% of persons. Of all participants, 29% (149/510) said that they used personal protective equipment (PPE; e.g. gloves, mask, gumboots) when handling animals. Participant responses regarding the use of PPE varied by occupation and gender (Tables 5 and 6). Butchers used PPE significantly more often than persons in other professions. However, they mostly used gumboots and aprons. Few butchers used gloves.

**Table 5:** Use of personal protective equipment (PPE) in persons with animal contact by occupation.

Type of PPE	Other Occupation (n-131)	Herdsmen (n = 32)	Farmers (n = 228)	Butchers (n = 115)	P-value*
**Any PPE**	15 (11%)	14 (44%)	28 (12%)	88 (77%)	**<0.01**
**Gloves**	4 (3%)	2 (6%)	2 (1%)	17 (15%)	**<0.01**
**Gumboots**	14 (11%)	14 (44%)	25 (11%)	84 (73%)	**<0.01**
**Masks**	1 (1%)	0	0	4 (3%)	**0.02**
**Eye**	1 (1%)	0	0	0	**0.4**
**Aprons**	3 (2%)	2 (6%)	0	69 (60%)	**<0.01**

*Compared by gender using chi-square

**Table 6:** Use of personal protective equipment in persons with animal contact by gender.

Type of PPE	Females n (% of Female)	Males n (% of Male)	P-value*	Total n (%)**
**Any PPE**	6 (4.2%)	143 (40%)	**<0.01**	149 (29%)
**Gloves**	2 (1.4%)	26 (7.2%)	**<0.01**	28 (5.5%)
**Gumboots**	4 (2.8%)	136 (38%)	**<0.01**	140 (27%)
**Masks**	0	5 (1.4%)	0.2	5 (0.98%)
**Eye**	0	1 (0.28%)	0.5	0.5
**Aprons**	0	77 (21%)	**<0.01**	77 (15%)

*Compared by gender using chi-square

**Total includes respondents who did not have a gender specified.

### Participant attitudes towards RVF

Most participants (88%; 501/567) believed that RVF exists; this finding did not vary by gender but did vary significantly by occupation. Compared to herdsmen (90%) and farmers (95%), fewer butchers (83%) and persons in other occupations (85%) (p<0.05) believed in the existence of RVF. The most common reason for not believing RVF exists is that people were not aware of an RVF case near Kabale (23%; 36/156); 28% (177/631) of all individuals were aware of an RVF case. Significantly more males (30%) than females (22%) had heard of an RVF case (p<0.05). Most people believed that they are at risk of getting RVF (69%; 448/651). Significantly more butchers identified themselves as being at risk of RVF (88%) compared to persons in other occupations (60%), farmers (68%), and herdsmen (79%) (p<0.01). Similarly a greater proportion of males (74%) identified themselves as being at risk of RVF compared to females (59%) (p<0.05). When asked about the treatment of RVF, most individuals said that they would seek care from modern medicine (91%; 579/636). Most participants said that they would interact with an RVF survivor (75%; 461/613) and welcome them back into their community (73%; 478/652).

## Discussion

Our study found that in the agricultural community of Kabale District, Uganda, most survey participants had heard of RVF, although few human cases had ever been identified in Uganda, and none had been identified since the 1960’s. Most of these participants received their information regarding RVF from the radio. This may have been in part due to local efforts to increase RVF awareness recently through radio announcements following the detection of the two human cases.

We found that although 90% of participants had heard of RVF, many did not recognize the most common signs and symptoms of RVF in humans and animals. The most common symptom recognized in both humans and animals was bleeding; however, this is a rare symptom of RVF. Survey participants may be confusing RVF symptoms with symptoms of Marburg virus, since a Marburg virus cluster was identified in Kabale district in 2012 and because Marburg can present with more hemorrhagic symptoms [25]. Our findings are similar to previous findings in other studies done in Kenya and Tanzania, despite differences in our study populations [19,22]. Compared to similar studies in Kenya and Tanzania, our study population had higher education; nearly 55% of our participants had secondary educations compared to 88% of participants having no formal education in the Kenya study by Abdi et al [19]. Further, Kenya has more documented RVF outbreaks than Uganda, with a particularly large outbreak in 2006–2007 that significantly impacted the Kenyan pastoralist economy [13]. In the Abdi study, most individuals (92%) recognized hemorrhage as an RVF symptom and less than 40% recognized headache, myalgias and visual changes as RVF symptoms in humans. Our study and the Abdi study suggest that even in settings where RVF is endemic or where formal education is prevalent, misconceptions about RVF exist.

Recognition of symptoms and signs in animals and humans is imperative to prevent the spread of disease, especially in a community such as Kabale, where 60% of survey participants own animals. During the 2007 RVF outbreak in Sudan, Hassan et al. noted that an RVF outbreak was only recognized after human cases were identified, indicating that improved awareness of the disease in animals should be emphasized [26]. When at-risk communities have a heightened awareness of RVF symptoms in animals they may be able to improve surveillance and identify an outbreak early. Jost et al. conducted focus groups with pastoralists in Kenya and Tanzania during the 2006–2007 RVF outbreak and found that pastoralists not only recognized changes in weather patterns and mosquito swarms but also recognized RVF signs among animals and humans [21].

Many participants (47%) said that they did not have an understanding of RVF transmission. Nearly a third (31%) of all of our participants thought that RVF could be transmitted by bodily contact and 66% of farmers thought that RVF could be transmitted by animal contact. Additionally, mosquito bites were an under-recognized mode of RVF transmission (this was also noted in the Abdi study) [19]. Interestingly, in another study in Kenya by Owange et al, pastoralists felt that mosquitoes were a very important risk factor for RVF transmission in cattle [20]. Although our study demonstrated that participants did not consider mosquitoes to be an important risk factor for RVF, participants said that they frequently slept under mosquito nets, likely due to concern for malaria. Improving education regarding other mosquito-borne diseases, including yellow fever and RVF, can further stress the importance of prevention against mosquito bites.

Studies of Ebola and Marburg survivors in Uganda and elsewhere have demonstrated that many survivors experience stigma within their community [27]. RVF, unlike Ebola and Marburg, cannot be transmitted from human to human. Our study demonstrated that stigma is not commonly associated with RVF and that most community members would interact with survivors and welcome them into their community.

The objective of our study was to conduct a rapid, timely KAP study in order to inform an educational campaign, and as a result our study had several limitations. Because our sampling strategy was convenience based, our survey participants may not accurately represent the true population in Kabale district. However, given that we interviewed over 650 participants in the region, focusing in part on high risk populations, we believe that our study is still very useful. Many of our questions were yes/no or multiple choice questions and we did not conduct any focus groups, therefore our results are mainly descriptive. Some of the questions regarding RVF symptoms were not specific to RVF and could overlap with other diseases including Marburg.

Since there is no currently approved RVF vaccine for use in humans [28] and no available veterinary RVF vaccine in Uganda, prevention of RVF is primarily through education. Educational programs should encourage the use of PPE during care of sick animals and slaughtering, discuss ways to decrease mosquito bites, and promote safe consumption of meats and dairy. In addition, heightened awareness of symptoms in humans and animals may lead to more rapid identification of outbreaks and may result in decreased transmission. Our study showed that RVF knowledge could be improved in Kabale district, Uganda.

### Development of RVF educational materials

Following the analysis of the survey, the MAAIF, Uganda Ministry of Health and CDC worked together to develop RVF educational materials targeting abattoir workers, farmers, herdsmen, and other community members (S2 Fig).

The posters emphasized that signs and symptoms of RVF more commonly include diarrhea, vomiting, and fever. These posters were created to target a low literacy audience using culturally appropriate messaging highlighting key findings from the KAP study, including disease transmission, signs and symptoms of RVF in humans and animals, and safe cooking practices [29]. For example, a poster targeting farmers and herdsmen contains images of symptomatic animals and humans and advises that farmers and herdsmen contact veterinary staff if their animal is ill. These materials have been translated to French for use in Niger after the identification of RVF cases in 2016 [30]. The CDC Viral Special Pathogens website also serves as a resource for information about previous outbreaks and RVF symptoms and diagnosis [31].

## Supporting information

S1 Checklist STROBE checklist. (DOCX)

S1 Fig Survey questionnaire. The questionnaire asked participants about knowledge, attitudes, and practice regarding Rift Valley Fever.
(PDF)

S2 Fig RVF educational materials. These materials were created to educate community members about signs and symptoms of Rift Valley Fever.
(PDF)
