# Supplementary material for: Rift Valley Fever: A survey of knowledge, attitudes, and practice of slaughterhouse workers and community members in Kabale District, Uganda
Source: PLoS Negl Trop Dis. 2018 Mar 5;12(3):e0006175. doi: 10.1371/journal.pntd.0006175 (PMC5860784; doi:10.1371/journal.pntd.0006175)
Supplement: S1 Fig — The questionnaire asked participants about knowledge, attitudes, and practice regarding Rift Valley Fever. (PDF) [file pntd.0006175.s002.pdf]

Uganda Ministry of Health

uvri Uganda Virus Research Institute

Form Approved OMB
No. 0920-1011
Exp. Date 03/31/2017

CONSENT FORM

Assessment of sero-prevalence of Rift Valley Fever virus in High risk populations to identify potential risk factors after an outbreak of Rift Valley Fever disease in Kabale 2016

Introduction and what we are doing

I am……………………………….. from Uganda Virus Research Institute/MoH/Kabale district. I am conducting an "Assessment of sero-prevalence of Rift Valley Fever virus in Kabale district to identify the extent of endemicity, risk factors for infection and identification high-risk areas and populations following and outbreak of Rift Valley Fever virus disease in Kabale 2016"

Purpose of the Assessment
This assessment will be important in informing the outbreak response to recent cases of Rift Valley Fever.

Procedures:
You have been chosen to participate in this assessment. In order to understand this condition better, you will be asked questions to assess your knowledge and daily activities. You will not be blamed for anything you say. You can choose to accept to participate or not to participate in this interview, but I would be happy if you agree. This interview will take a maximum of 20 minutes. You will also be asked to provide a blood sample of maximum 4ml. If you and your household care for animals, we would also like to ask some questions and collect blood samples from some of these animals. The samples will be tested in the coming months for Rift Valley fever virus, which is circulating in Kabale district and possibly surrounding districts. We are looking to see if you have been previously infected with this virus. We may also use or store this sample to test for previous infection with other infectious diseases, such as Marburg and Ebola viruses, CCHF, Rift Valley Fever and Yellow fever, or other zoonotic pathogens.

Risks: The assessment has no harm but only takes respondents' time to participate in answering the questions. You may experience pain for short time during blood sample collection

Benefits: Taking part in this assessment may not provide you with direct benefits however you and your community members may benefit in future from the information that will be learned from this assessment.

Incentives: You will not be provided with any incentives to take part in this assessment

Confidentiality: The information obtained here will be confidential and will only be used for outbreak response. Information collected about you will be stored in a file that does not bear your name but only a code and kept under lock and key and will not be divulged to anyone except the assessment team and sponsors

Right to refuse or to withdraw: You do not have to take part in this assessment if you do not wish, and refusing to participate will not affect you or your family. You may stop participating in this assessment at any time that you wish without losing any of your rights

Who to contact: If you have any questions regarding this assessment, you may ask now or later. If you wish to later, you may contact the following number Dr. Luke Nyakarahuka, Uganda Virus Institute, Entebbe**,** 0776970840, 0752970840

REC Contact: In case of any ethical issues arising out of this assessment, contact the Research and Ethics Committee Chairman at Telephone Number 0414321962, Uganda Virus Research Institute

Certificate of consent to the assessment
I confirm that I have read the information sheet above concerning the assessment and have had the opportunity to ask questions and have had these answered satisfactorily. I understand that my participation is voluntary and I am free to withdraw consent at any time, without giving a reason, without my legal rights being affected. I understand that the blood sample collected from me will be tested for Viral hemorrhagic fevers such as Marburg and Ebola viruses, CCHF, Rift Valley and Yellow fever, or other zoonotic pathogens. I also know that the samples will be retained at UVRI for future testing of any other infectious agents. I understand that data collected may be looked at by responsible representatives from the UVRI for the purposes of monitoring and auditing to ensure that the assessment is being conducted properly. I give permission for these individuals to have access to relevant information and I agree to participate in the assessment

Name of participant…………….…………………………… Signature/Thumbprint……………………………….. Date (d/m/y)……………..

Name of Witness_______________________________________ Signature_____________________________ Date (d/m/y)________

Name of data Collector__________________________________ Signature_____________________________ Date (d/m/y)________

END OF CONSENT FORM

Public reporting burden of this collection of information is estimated to average 30 minutes per response, including the time for reviewing instructions, searching existing data sources, gathering and maintaining the data needed, and completing and reviewing the collection of information. An agency may not conduct or sponsor, and a person is not required to [illegible]

Uganda Ministry of Health

uvri Uganda Virus Research Institute

Form Approved OMB
No. 0920-1011
Exp. Date 03/31/2017

CONSENT FORM

Assessment of sero-prevalence of Rift Valley Fever virus in High risk populations to identify potential risk factors after an outbreak of Rift Valley Fever disease in Kabale 2016

Introduction and what we are doing

I am……………………………….. from Uganda Virus Research Institute/MoH/Kabale district. I am conducting an "Assessment of sero-prevalence of Rift Valley Fever virus in Kabale district to identify the extent of endemicity, risk factors for infection and identification high-risk areas and populations following and outbreak of Rift Valley Fever virus disease in Kabale 2016"

Purpose of the Assessment
This assessment will be important in informing the outbreak response to recent cases of Rift Valley Fever.

Procedures:
You have been chosen to participate in this assessment. In order to understand this condition better, you will be asked questions to assess your knowledge and daily activities. You will not be blamed for anything you say. You can choose to accept to participate or not to participate in this interview, but I would be happy if you agree. This interview will take a maximum of 20 minutes. You will also be asked to provide a blood sample of maximum 4ml. If you and your household care for animals, we would also like to ask some questions and collect blood samples from some of these animals. The samples will be tested in the coming months for Rift Valley fever virus, which is circulating in Kabale district and possibly surrounding districts. We are looking to see if you have been previously infected with this virus. We may also use or store this sample to test for previous infection with other infectious diseases, such as Marburg and Ebola viruses, CCHF, Rift Valley Fever and Yellow fever, or other zoonotic pathogens.

Risks: The assessment has no harm but only takes respondents' time to participate in answering the questions. You may experience pain for short time during blood sample collection

Benefits: Taking part in this assessment may not provide you with direct benefits however you and your community members may benefit in future from the information that will be learned from this assessment.

Incentives: You will not be provided with any incentives to take part in this assessment

Confidentiality: The information obtained here will be confidential and will only be used for outbreak response. Information collected about you will be stored in a file that does not bear your name but only a code and kept under lock and key and will not be divulged to anyone except the assessment team and sponsors

Right to refuse or to withdraw: You do not have to take part in this assessment if you do not wish, and refusing to participate will not affect you or your family. You may stop participating in this assessment at any time that you wish without losing any of your rights

Who to contact: If you have any questions regarding this assessment, you may ask now or later. If you wish to later, you may contact the following number Dr. Luke Nyakarahuka, Uganda Virus Institute, Entebbe**,** 0776970840, 0752970840

REC Contact: In case of any ethical issues arising out of this assessment, contact the Research and Ethics Committee Chairman at Telephone Number 0414321962, Uganda Virus Research Institute

Certificate of consent to the assessment
I confirm that I have read the information sheet above concerning the assessment and have had the opportunity to ask questions and have had these answered satisfactorily. I understand that my participation is voluntary and I am free to withdraw consent at any time, without giving a reason, without my legal rights being affected. I understand that the blood sample collected from me will be tested for Viral hemorrhagic fevers such as Marburg and Ebola viruses, CCHF, Rift Valley and Yellow fever, or other zoonotic pathogens. I also know that the samples will be retained at UVRI for future testing of any other infectious agents. I understand that data collected may be looked at by responsible representatives from the UVRI for the purposes of monitoring and auditing to ensure that the assessment is being conducted properly. I give permission for these individuals to have access to relevant information and I agree to participate in the assessment

Name of participant……………..…………………………… Signature/Thumbprint……………………………….. Date (d/m/y)……………..

Name of Witness____ Signature_______________________________ Date (d/m/y)________

Name of data Collector____________________________________ Signature_______________________________ Date (d/m/y)________

END OF CONSENT FORM

Public reporting burden of this collection of information is estimated to average 30 minutes per response, including the time for reviewing instructions, searching existing data sources, gathering and maintaining the data needed, and completing and reviewing the collection of information. An agency may not conduct or sponsor, and a person is not required to respond to a collection of information unless it displays a currently valid OMB control number. Send comments regarding this burden estimate or any other aspect of this collection of

Uganda Ministry of Health | uvri Uganda Virus Research Institute

Form Approved OMB
No. 0920-1011
Exp. Date 03/31/2017

Questionnaire

Date: ____/____/_____ (D, M, Yr) **Sample ID**______________________________________ **Participant classification**__________________

Form Completed by

Name: _____________________ Position: ________________District: ______________________

Phone Number/email: ____

Section 1. Assessment Participant Information

ID Number: ____________________ Name:_______________________________________ Age: _________Gender: ☐ Male ☐ Female.

Village/Town: __________________ Parish: _____________________Sub-County: _______________________

District: _________________Nationality: _____________ Marital status: ☐ Married ☐ Single ☐ Widowed

GPS Coordinates____

Section 2. Epidemiological Risk Factors and Exposures

1. Education level: ☐None ☐Primary ☐Secondary ☐Post-Secondary ☐Other, specify________________________________
2. Primary Occupation: ☐ Farmer ☐ Herdsman ☐ Housewife ☐ Student ☐ Child ☐ Animal Health worker ☐ Butcher ☐ Trader ☐Other, please specify occupation____
3. Do you or your household own domestic animals ☐ Yes ☐ No **(If NO, skip to #5)**
4. If yes, which ones: ☐ goats (n=____) ☐ sheep (n=____) ☐ Cattle (n=____) ☐ Pigs (n=____) ☐ Poultry (n=____) ☐ Dogs (n=____) ☐ cats (n=____) ☐ others, specify__________________________
5. In the past year, have you had contact with domestic animals ☐ Yes ☐ No **(If NO, skip to #8)**
6. If yes, which domestic animals have you had contact with *(tick all that apply)* ☐ goats ☐ Cattle ☐ Pigs ☐ Poultry ☐ Dogs ☐others specify_________________
7. If yes, which type of contact *(tick all that apply)* ☐ during milking ☐during grazing ☐grooming ☐caring sick animals ☐slaughtering/skinning/butchering ☐ preparing or handling raw meat ☐sleeping near animals ☐others, specify_________
8. In the past one year, have you drunk raw milk ☐ Yes ☐ No
9. In the past one year, have you eaten raw/uncooked meat ☐ Yes ☐ No
10. In the past one year, have you been involved in processing(handling or preparing) meat ☐ Yes ☐ No
11. In the past one year, have you been involved in Fetus/*abortus* disposal ☐ Yes ☐ No
12. In the past one year, have you assisted livestock(cattle, sheep and goats) to give birth ☐ Yes ☐ No
13. In the past one year, have you been involved in butchering livestock ☐ Yes ☐ No
14. In the past one year, have you seen wild animals in this village ☐ Yes ☐ No **(If NO, skip to #16)**
15. If yes, which ones: ☐ monkeys ☐ bats ☐ antelopes ☐ wild pigs ☐ others specify_______
16. In past one year , have you any contact with wild animals ☐ Yes ☐ No **(If NO, skip to #19)**
17. If yes, which wild animals do you usually get in contact with (tick all that apply) ☐Bats ☐monkeys ☐wild Pigs ☐wild birds ☐rodents ☐antelopes ☐others specify___________________________
18. If yes, which type of contact ☐during hunting ☐accidental ☐slaughtering ☐other specify__________________________
19. In past one year , have you done hunting ☐Yes ☐No ☐Unk, **(If NO, skip to #22)**
20. If yes, how often do you do hunting________________________________
21. Which animals are usually hunted________________________________
22. In past one year , have you had contact with dead wild animals ☐ Yes ☐ No ☐ Unk
23. Do you use PPE when handling animals ☐ Yes ☐ No ☐ Unkn **(If NO, skip to #25)**
24. If yes, which ones ☐ gloves ☐ gumboots ☐masks ☐Eye protection ☐Aprons/ovals ☐others specify_____________
25. Have you eaten wild meat in the past one year ☐ Yes ☐ No ☐ Unk **(If NO, skip to #26)**
26. If Yes, which species____________________
27. Have you traveled outside your home or village/town in the past one year, ☐ Yes ☐ No ☐ Unkn **(If NO, skip to #28)**
28. If yes, specify location and date: ____
29. Did you ever suffer from undiagnosed fever or illness in the past one year, ☐ Yes ☐ No ☐ Unkn, **(If NO, skip to #30)**
30. If yes, did you seek medical attention: ☐ Yes ☐ No

Public reporting burden of this collection of information is estimated to average 30 minutes per response, including the time for reviewing instructions, searching existing data sources, gathering and maintaining the data needed, and completing and reviewing the collection of information. An agency may not conduct or sponsor, and a person is not required to respond to a collection of information unless it displays a currently valid OMB control number. Send comments regarding this burden estimate or any other aspect of this collection of [illegible]

Uganda Ministry of Health

uvri Uganda Virus Research Institute

Form Approved OMB
No. 0920-1011
Exp. Date 03/31/2017

31. Have you heard of someone acutely ill in the last one year with unexplained fever or diagnosis?
    ☐ Yes ☐ No ☐ Unkn
32. Have you had *el nino* (have you had more rainfall than usual*)* rains in the last one year? ☐ Yes ☐ No ☐ Unkn
33. Have you had flooding in this sub-county in past one year? ☐ Yes ☐ No ☐ Unkn **(If NO, skip to #34)**
34. If yes, which months do you get flooding____
35. In the past 1 year, have you seen more mosquitoes than usual in this village? ☐ Yes ☐ No
36. In the past year, have you been bitten by more mosquitoes than usual? ☐ Yes ☐ No
37. In the past year, have you been using a mosquito net? ☐ Yes ☐ No ☐ Unkn **(If NO, skip to #38)**
38. If yes/No, why____
39. In past year, have you sprayed animals against external parasites? ☐ Yes ☐ No ☐ Unkn
40. Have you done indoor spraying against mosquitoes in the last one year? ☐ Yes ☐ No ☐ Unkn
41. Have you been the forest/bush in past one year ☐ Yes ☐ No ☐ Unk

Knowledge & Attitude Questions

42. Have you heard about Rift Valley Fever Disease? ☐Yes ☐No ☐Unk **(If NO, skip to #43)**
43. If yes, from whom: ☐Health worker ☐Radio ☐community leaders ☐others, specify______________________________
44. Do you know the signs and symptoms of RVF virus disease in humans? ☐Yes ☐No ☐ Unk **(If NO, skip to #45)**
45. If yes, what are some signs and symptoms? ☐bleeding ☐fever ☐vomiting ☐diarrhea ☐others specify______________
46. Do you know the signs and symptoms of RVF virus disease in animals? ☐Yes ☐No ☐Unk **(If NO, skip to #47)**
47. If yes, what are some signs and symptoms in animals? ☐bleeding ☐fever ☐nasal discharge ☐diarrhea ☐abortion
    ☐reduced milk production ☐prostration ☐loss of appetite ☐others, specify______________________________
48. Do you know who to contact in case you see a suspect case of RVF disease? ☐ Yes ☐ No ☐ Unk
49. Do you believe RVF disease really exists? ☐ Yes ☐ No ☐ Unk **(If YES, skip to #50)**
50. If no, why____
51. Have you heard of any survivor of RVF disease? ☐ Yes ☐ No ☐ Unk
52. Would you relate/interact with a survivor of RVF disease ☐ Yes ☐ No ☐ Unk
53. Would you welcome someone back into their community/neighborhood after a neighbor has recovered from RVF?
    ☐Yes ☐No ☐Unk **(If YES, skip to #54)**
54. If no, why? ☐fear of contracting disease ☐fear of stigma from community ☐others specify_______________________
55. Do you know how RVF disease is transmitted? ☐ Yes ☐ No ☐ Unk **(If NO, skip to #57)**
56. If yes, how? ☐ body contact sick person ☐ through air ☐ through needle pricks ☐ contact with animals ☐contact with dead person ☐ contact with body fluids of sick person ☐biting mosquitoes(insects) ☐ others specify________________
57. If transmission through animals, which ones? ☐ goats ☐ Cattle ☐ Sheep ☐ Poultry ☐ Dogs
    ☐ monkeys ☐ bats ☐ antelopes ☐ wild pigs ☐ others specify_____________
58. How do you think you can protect yourself from acquiring RVF disease?
    ☐ vaccination ☐ avoiding contact with animals ☐ traditional medicine ☐ avoiding sick people
    ☐ sleeping in a mosquito net ☐others specify____
59. How do you think RVF disease can best be healed or treated?
    ☐traditional medicine ☐spiritual healing ☐Modern medicine ☐Herbal medicine ☐others specify_________________
60. Do you think you are at risk of contracting RVF virus disease? ☐ Yes ☐ No ☐ Unk
61. If yes/no, why____
62. Do you know how RVF disease can be prevented ☐ Yes ☐ No ☐ Unk **(If NO, skip to end of interview)**
63. If yes, how____

Thank you for your Time

End of Interview

Section 3. Specimen Information

Specimen identification number:___________________

Specimen collection date:___/___/______ (MM/DD/YYYY)

Laboratory testing date:___/___/______ (MM/DD/YYYY)

Results/Titer level: IgM _____________ IgG _____________

Public reporting burden of this collection of information is estimated to average 30 minutes per response, including the time for reviewing instructions, searching existing data sources, gathering and maintaining the data needed, and completing and reviewing the collection of information. An agency may not conduct or sponsor, and a person is not required to respond to a collection of information unless it displays a currently valid OMB control number. Send comments regarding this burden estimate or any other aspect of this collection of [illegible]
