# Supplementary material for: Rift Valley Fever: A survey of knowledge, attitudes, and practice of slaughterhouse workers and community members in Kabale District, Uganda
Source: PLoS Negl Trop Dis. 2018 Mar 5;12(3):e0006175. doi: 10.1371/journal.pntd.0006175 (PMC5860784; doi:10.1371/journal.pntd.0006175)
Supplement: S2 Fig — These materials were created to educate community members about signs and symptoms of Rift Valley Fever. (PDF) [file pntd.0006175.s003.pdf]

WHAT YOU NEED TO KNOW ABOUT
RIFT VALLEY FEVER (RVF)

WITH GOOD KNOWLEDGE AND GOOD INFORMATION, WE CAN PREVENT PEOPLE AND ANIMALS FROM GETTING RIFT VALLEY FEVER.

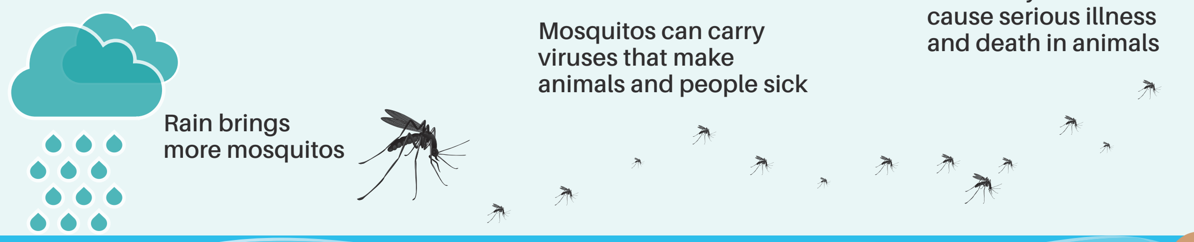

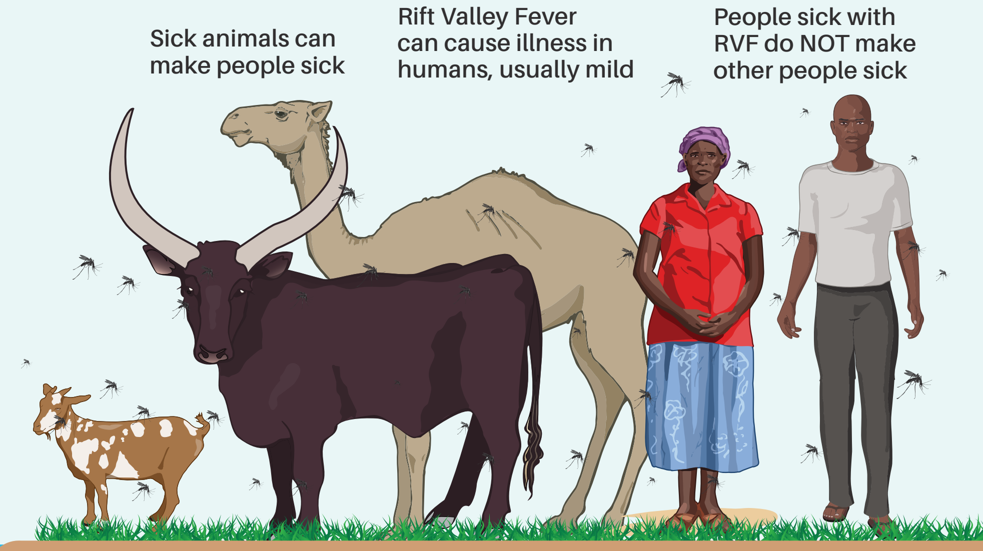

SIGNS OF A RIFT VALLEY FEVER OUTBREAK

- Many unexpected pregnancy losses in goats, sheep, and cattle
- Stillbirths and births of weak animals
- Illness and death of young livestock (less than one year of age)

SYMPTOMS OF RVF IN ANIMALS

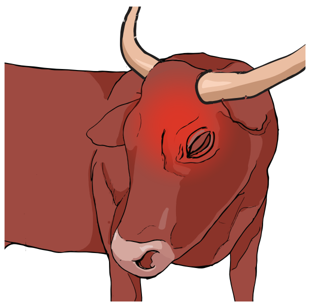

Animal may feel hot to the touch

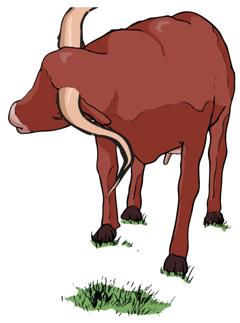

Not eating: an animal that suddenly stops eating may be sick

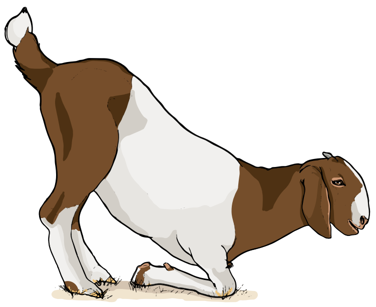

Weakness: animal may not be able to stand or move around

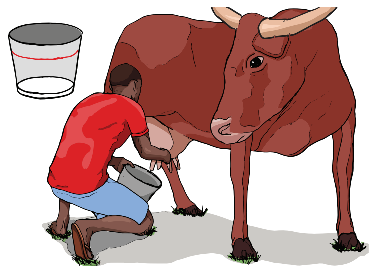

A sudden decrese or drop in milk production

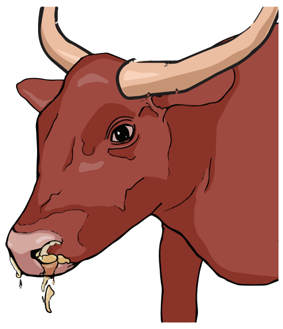

Water from nose: a clear or blood colored liquid may drip from the nose

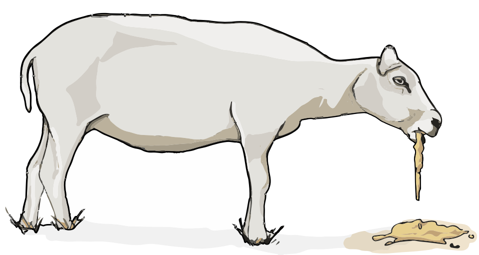

Vomiting

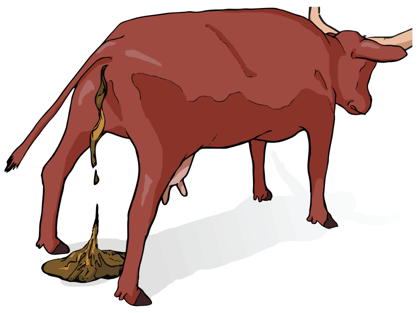

Diarrhea: the animal may have running stomach. May be bloody or not.

Once animals recover from Rift Valley Fever, they are no longer able to infect people.

RVF IN PEOPLE

Those at greatest risk of getting Rift Valley Fever are people with contact with sick animals, including:

- Animal health workers
- Herders, other people who take care of sick animals
- Abattoir workers and people involved in slaughtering sick animals
- People who may touch the bodies of sick or dead animals
- Those who may handle uncooked meat or drink raw milk of sick animals

Typically, people infected with Rift Valley Fever recover 2-7 days after mild illness; however, a small number of people develop much more severe symptoms.

Signs of serious illness in people include: vomiting, diarrhea, muscle or joint pain, intense fatigue, abdominal pain, and unexplained bleeding.

One person cannot infect another person with Rift Valley Fever.

Seeking care early is an important way to help your chances of survival if you become seriously ill. Although there is no cure for RVF, there is supportive care, such as transfusions and IV fluids that can help save lives.

WHEN ANIMALS OR FAMILY MEMBERS ARE SICK:

If you notice an abnormal number of spontaneous abortions, stillbirths or increased numbers of of premature deaths in animals, report it to veterinary services for coordination and reporting of testing, and, if necessary, inform the Ministry of Health.

Contact: ____________________

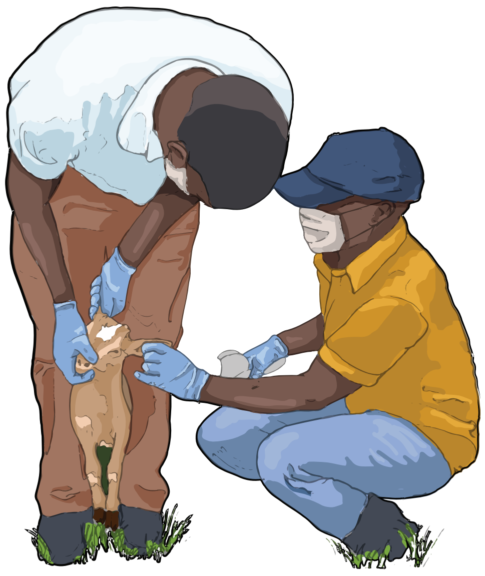

When testing and drawing samples from animals, veterinarians should follow the recommendations below. To avoid contact live with bodily fluids sick or dead animals, or products of abortion, veterinarians should wear gloves, boots, long sleeves, and a face shield to protect against splashing.

U.S. Department of Health and Human Services
Centers for Disease Control and Prevention

CS265393D

WHAT YOU NEED TO KNOW ABOUT RIFT VALLEY FEVER (RVF)

WITH GOOD KNOWLEDGE AND GOOD INFORMATION, WE CAN PREVENT PEOPLE AND ANIMALS FROM GETTING RIFT VALLEY FEVER.

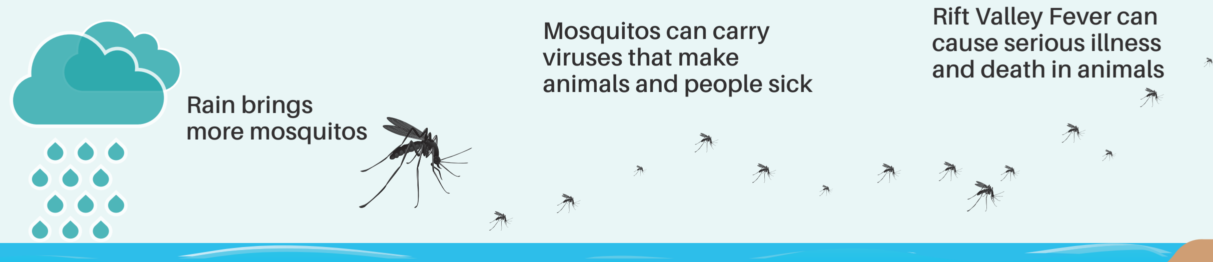

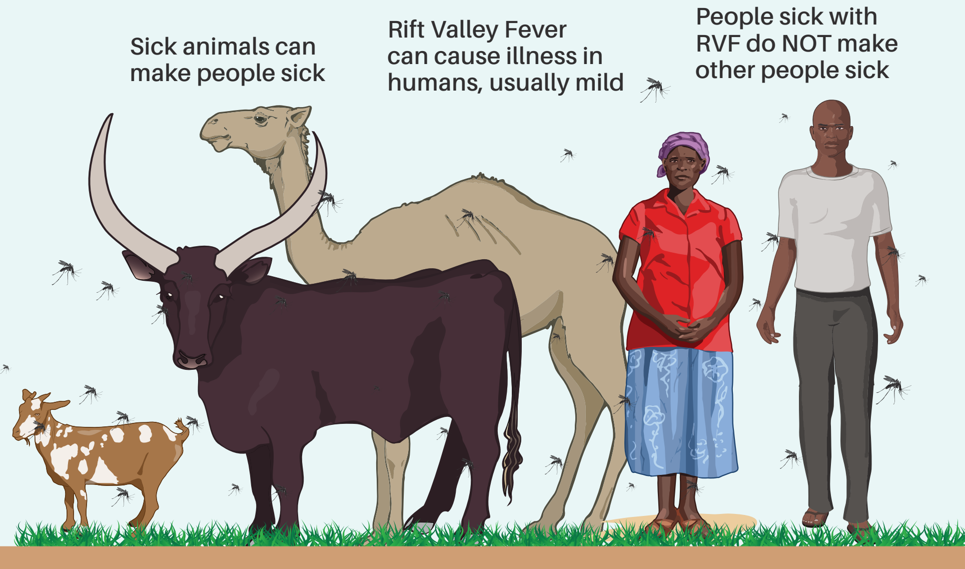

FOR HERDERS, FARMERS, BUTCHERS AND ABATTOIR WORKERS

With Rift Valley Fever, usually animals such as goats, cattle, and sheep become sick first and then humans become sick. Humans can become sick after they have been in contact with sick animals. RVF does not spread from one person to another person.

RIFT VALLEY FEVER IN ANIMALS

RVF is very serious in animals. It causes aborted pregnancies, or being born dead, and increased death in young animals.

OTHER SYMPTOMS IN ANIMALS INCLUDE:

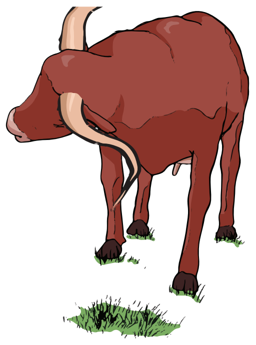
Loss of appetite

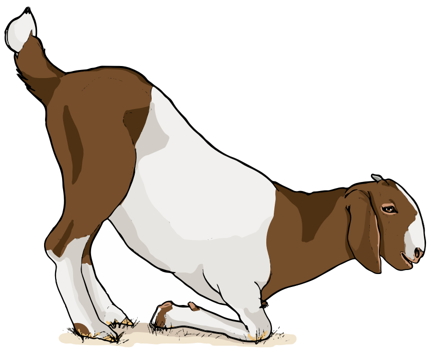
Weakness

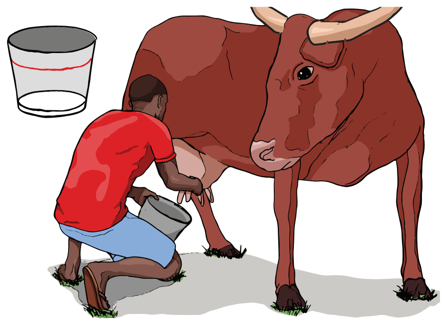
Decreased milk production

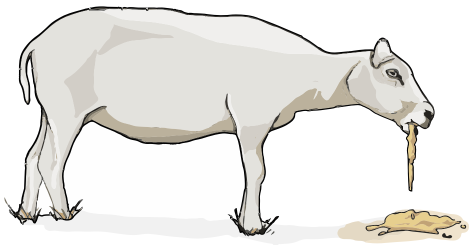
Vomiting

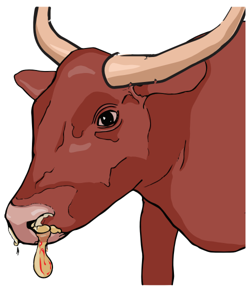
Nasal discharge

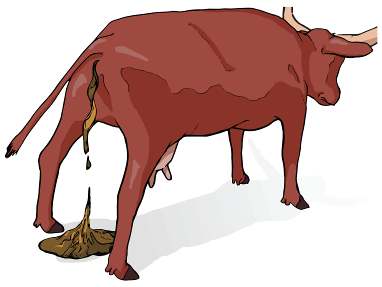
Diarrhea

Tell your village leaders and health officials if you notice these signs and symptoms. Once animals recover from Rift Valley Fever, they are no longer able to infect people.

RIFT VALLEY FEVER IN PEOPLE

Most people with RVF have no symptoms at all or only mild illness.
RVF does not spread from one person to another person.

People who do become ill might experience:

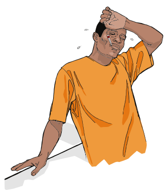
Fever

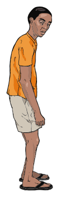
Weakness

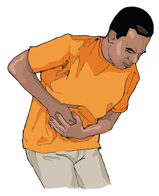
Stomach pain

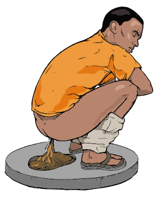
Diarrhea

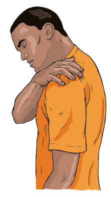
Joint pain

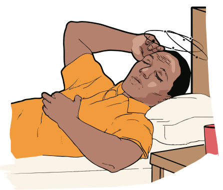
Dizziness

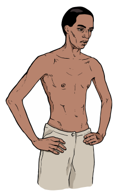
Weight loss

Typically, patients recover 2-7 days after onset of illness if treatment is sought early.
In a small number of patients, more serious illness can happen, including symptoms of:

- Vomiting
- Bleeding (blood in vomit, blood in diarrhea, bleeding gums, red eyes)
- Headaches, coma, or seizures
- Blurred vision, reddening of the eyes, decreased vision, and sometimes loss of vision

WHEN ANIMALS OR FAMILY MEMBERS ARE SICK:

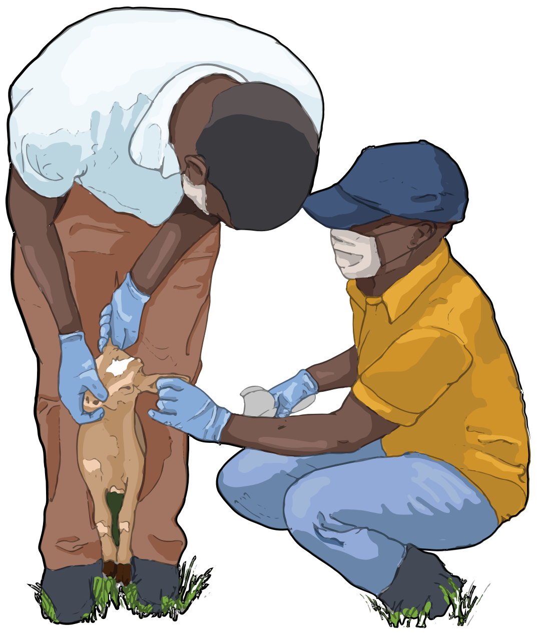

- Report sick livestock, abortions, and unexpected deaths to the local veterinarian.
- Do not handle or bury dead livestock. Call the local veterinarian to dispose of the body correctly.

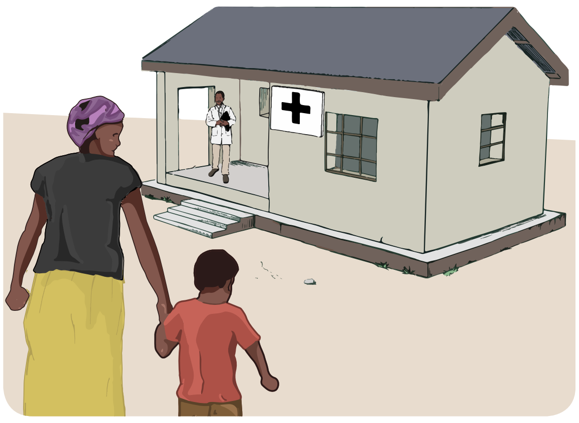

- If you feel sick, visit the local clinic or hospital.
- Seek care early to help your chances of survival if you become seriously ill.

U.S. Department of Health and Human Services
Centers for Disease Control and Prevention

CS265393C

WHAT YOU NEED TO KNOW ABOUT
RIFT VALLEY FEVER (RVF)

WITH GOOD KNOWLEDGE AND GOOD INFORMATION, WE CAN PREVENT PEOPLE AND ANIMALS FROM GETTING RIFT VALLEY FEVER.

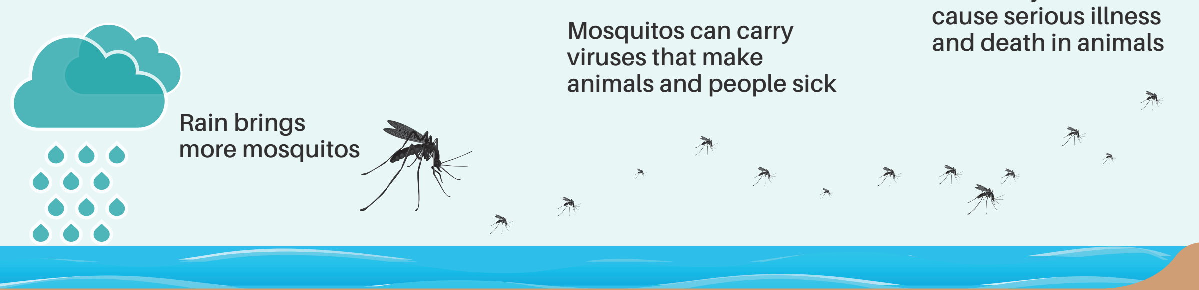

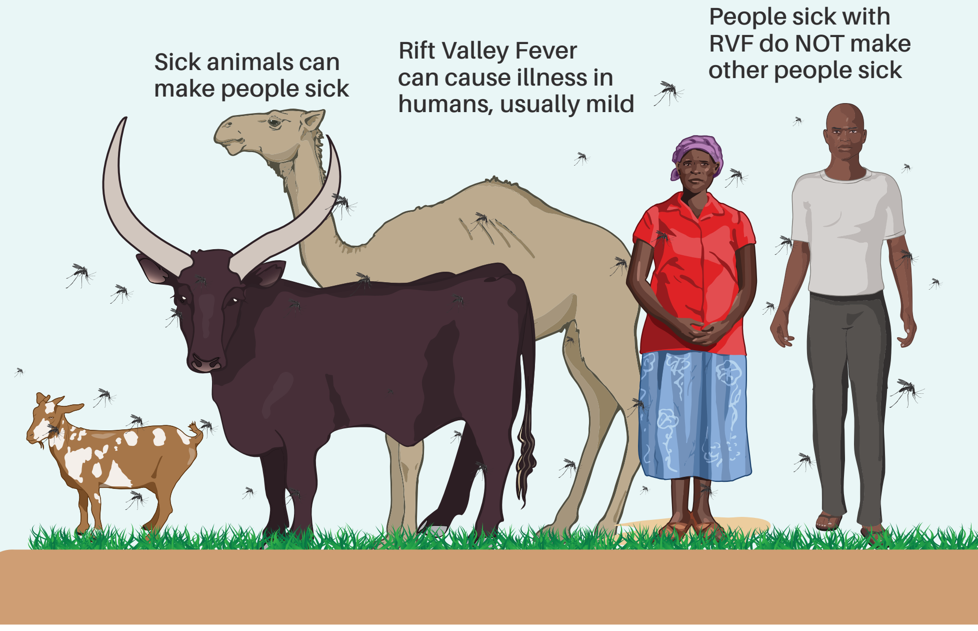

HOW TO PREVENT RVF

1. AVOID CONTACT WITH SICK ANIMALS AND MEAT OR MILK FROM SICK ANIMALS

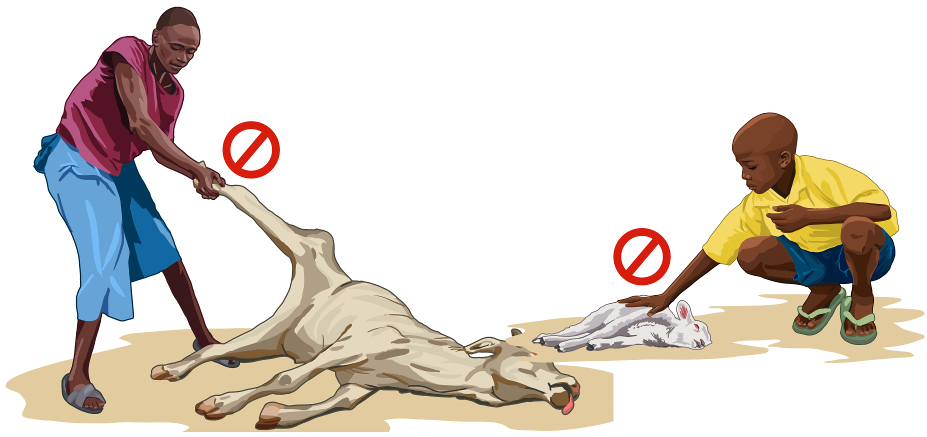

WARNING

Call veterinarians if your animals are sick or have had an abortion

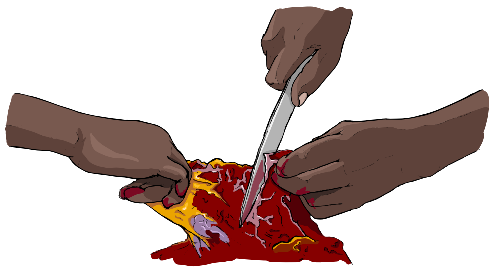

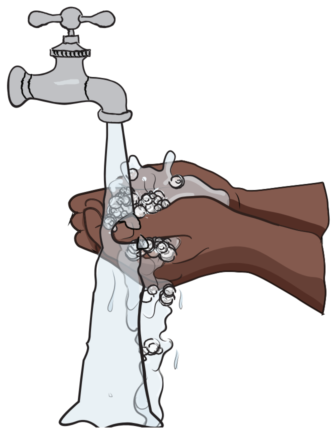

Wash your hands after touching raw meat or milk

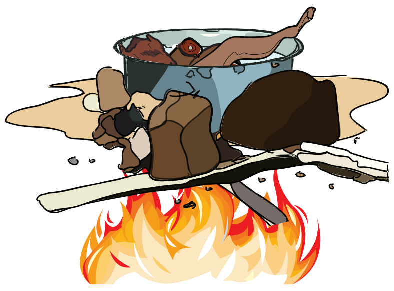

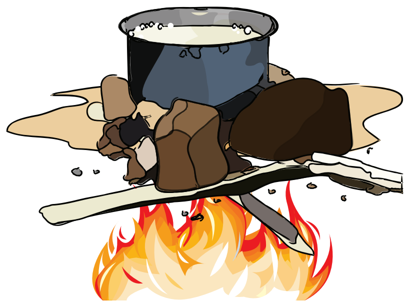

Cook meat thoroughly; boil raw milk2.

2. AVOID MOSQUITO BITES

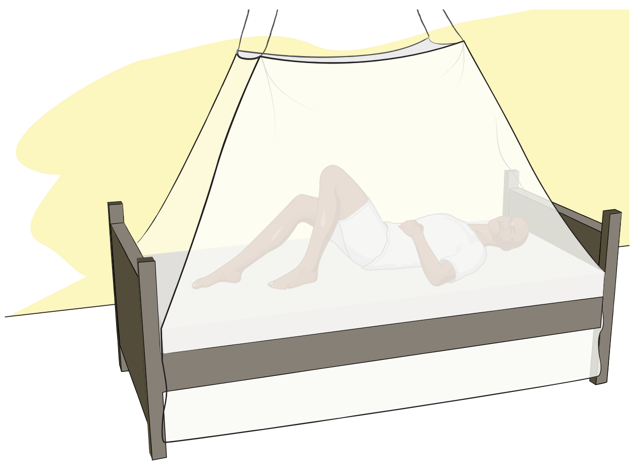

Use bednets to protect you from getting diseases from mosquitos like Rift Valley Fever, malaria, and Yellow fever

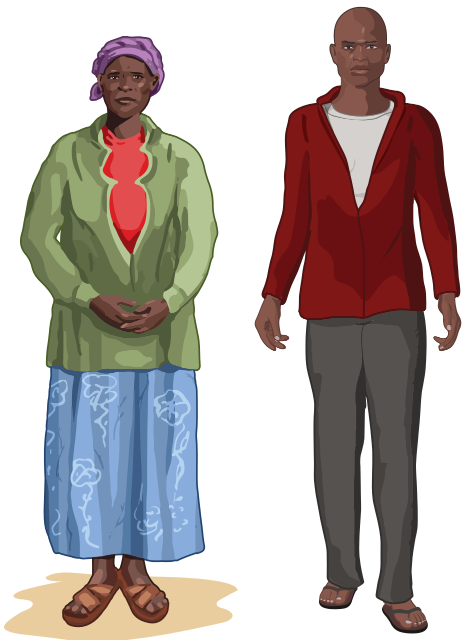

Wear long clothing to cover the body

WHEN ANIMALS OR FAMILY MEMBERS ARE SICK:

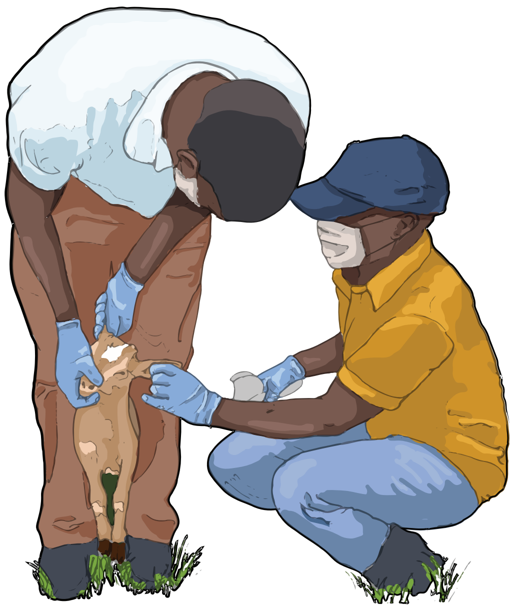

- Report sick livestock, abortions, and unexpected deaths to the local veterinarian.
- Do not handle or bury dead livestock. Call the local veterinarian to dispose of the body correctly.

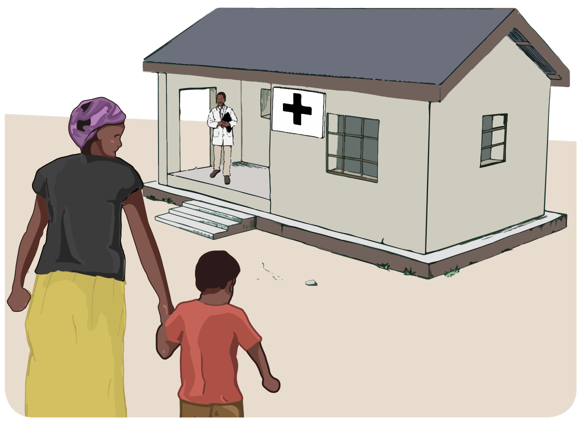

- If you feel sick, visit the local clinic or hospital.
- Seek care early to help your chances of survival if you become seriously ill.

U.S. Department of Health and Human Services
Centers for Disease Control and Prevention

CS265393B
